# Perceived Multi-Level Diabetes Protective and Risk Factors Associated with Food and Dietary Acculturation Patterns Among East African Immigrants in the United States

**DOI:** 10.3390/healthcare14203430

**Published:** 2026-10-14

**Authors:** Habiba Ibrahim, Angela Ekwonye, Salma Ibrahim, Nimo Mohamed, Muna Shidane, Artam Enayat, Melika Espahbodi

**Affiliations:** 1 Social Work Department, St. Catherine University, St Paul, MN 55105, USA; nomohamed380@stkate.edu (N.M.); aenayat807@stkate.edu (A.E.); mespahbodi464@stkate.edu (M.E.); 2 Public Health Department, St. Catherine University, St Paul, MN 55105, USA; amekwonye034@stkate.edu (A.E.); smibrahim689@stkate.edu (S.I.); 3 International Studies Department, St. Catherine University, St Paul, MN 55105, USA; mashidane187@stkate.edu

**Keywords:** immigrants, East African immigrants, food acculturation, dietary changes, diabetes perceived risk and protective factors, socioecological, multi-level factors

## Abstract

**Background:** Upon arrival in a new country, immigrants have lower body mass index (BMI), and are less likely to be overweight and obese than natives. However, longer residence is associated with higher BMI, partly attributed to adoption of unhealthy diets and dietary habits. Overweight and obesity (primary risk factors for diabetes) have been associated with food acculturation—the process by which immigrants adopt food practices of their new country. In particular, unfamiliar food cultures and food purchasing environment, lack of knowledge on how to prepare and incorporate new foods in traditional diets, perceptions towards food in new countries, all limit immigrants’ food choices and access. However, very few food acculturation studies have focused on African immigrants, specifically from eastern Africa. Yet, this group reports high rates of overweight, obesity, and higher prevalence of diabetes and pre-diabetes. Using the socioecological framework, this study explored the multi-level facilitators and barriers associated with food and dietary acculturation patterns and perceived diabetes-related health risks among East African immigrants and refugees in the Twin Cities Metro Area, Minnesota, United States. **Methods:** Six semi-structured focus groups were conducted with 36 Somali and Ethiopian women in the summer and fall of 2025. Participants represented Somali, Oromo and Amharic speakers. Only women were recruited with the assumption that women are the primary food decision makers and preparers in these communities. Therefore, the 36 participants provided insight into their households’ dietary practices and food decision-making processes. All focus group discussions were audio recorded to ensure accuracy and authenticity of participants’ narratives, then directly translated into English within a few days of being conducted. Each transcript was cross-checked by at least two team members. Data were analyzed thematically using Braun and Clarke’s approach: codes, categories, themes, and subthemes were iteratively refined through collaborative team review and organized within the socioecological framework. **Results:** The analysis identified two overarching themes at the individual, family, community and structural levels. Attachment to traditional and ethnic foods functioned as important cultural and health-related anchors. Participants shared their preference for ethnic foods as healthier, more natural, tastier and central to identity and continuity after migration. Sustaining healthy dietary practices at the individual and family levels was shaped by complex factors such as: work and caregiving demands, stress, time constraints, and changing family dynamics and roles. Intergenerational differences were especially salient. Children adopted American foods faster, increasing reliance on fast and convenience foods, creating household tensions, and sometimes requiring separate meal preparations. Community and structural factors further shaped dietary choices. High costs, limited availability of cultural foods, distance to ethnic stores, and transportation barriers constrained access, while highly processed and sugary foods were easily accessible. Participants also described reduced routine physical activity and migration-related stress as contributors to perceived diabetes risk. Ethnic grocery stores, halal markets, community networks, recipe adaptation, and the continued consumption of traditional foods were perceived as protective resources amidst these challenges. Overall, the findings appear to demonstrate that food and dietary acculturation are not a linear process in which immigrants shift from a traditional to a Western-based diet. Rather, it is shaped by complex interactions across individual, family, community, and structural factors. The study limitations, and implications for practice and research are discussed in the paper.

## 1. Introduction and Background

### 1.1. Immigrants Populations and Health Transitions upon Migration

The United States has a sizable and rapidly growing foreign-born population, which accounted for more than15% of the U.S. population by the end of 2024 [1,2]. This number gets even higher when immigrant households are considered including U.S. born children accounting for 27% of the overall U.S. population [3]. Although this group is all categorized as foreign-born or immigrants, they are very diverse communities in terms of country of origin, race/ethnicity, language, culture, and religion [1,2]. It is important to note that this diversity influences differences in health outcomes as they resettle and adjust to life in the United States.

Upon arrival, immigrants have shown initial health advantages in certain health outcomes commonly described as the “Healthy Immigrant Effect” (HIE) [4]. Specifically, immigrants have lower body mass index (BMI) and are less likely to be overweight and obese than native-born Americans [5,6]. However, this initial advantage declines with increased duration of residence, as they encounter new food environments, reduced physical activity, chronic stress, and barriers to preventive and culturally responsive healthcare [5,7,8]. Thus, studies show an increasing prevalence of diabetes and its risk factors, such as obesity and overweight [9,10,11,12,13].

Furthermore, the trajectory of this change is not uniform, as differences in race, ethnicity, and migration context shape patterns of diabetes risk across immigrant populations [14]. These factors interact to influence dietary behaviors and increase vulnerability to chronic diseases [15]. Therefore, understanding these transitions is important for identifying at-risk populations and designing targeted interventions.

### 1.2. East African Immigrants and Minnesota Context

African immigrants are among the rapidly growing immigrant groups in the United States [16]. Their number has more than doubled since 2000, increasing from 2.4 million to almost six million [16]. In Minnesota, and specifically Minneapolis–St. Paul metropolitan area (Twin Cities metro area), where this study was conducted, is home to a large and growing immigrant community. Recent census data reports that about 13.4% of the Twin Cities population are foreign-born [17,18]. It also hosts one of the largest East African Immigrants communities in the nation, primarily from Somalia and Ethiopia [18]. Formal reports estimate their populations statewide at around 80,000 and 40,000, respectively [17,19]. Informal reports put the size of the East African Immigrants at over 110,000 [20]. This demographic concentration provides a critical context for examining health disparities impacting these communities.

While research specific to the East African Immigrants is scant, emerging evidence indicates elevated rates of overweight, obesity, prediabetes, diabetes, coupled with low diabetes health literacy among these communities [21,22,23,24,25]. For example, a study conducted in a large midwestern regional health network revealed that Somali patients exhibited higher rates of obesity (34.6 vs. 32.1%; *p* = 0.047), prediabetes (21.3 vs. 17.2%; *p* < 0.02), and diabetes (12.1 vs. 5.3%; *p* = 0.0001) compared to non-Somali patients [23], with growing concern about cardiometabolic risk across other East African groups as well [21,22,23,24,25].

Evidence from international immigrant populations further supports elevated cardiometabolic risk among East African and other immigrant groups, with studies documenting increased prevalence of obesity, diabetes, and cardiovascular risk across diverse resettlement contexts [26,27,28,29]. Studies that assessed African immigrants in high-income countries similarly found high prevalence of diabetes and related risk factors among these populations with estimates suggesting rates comparable to or exceeding those of native populations [26,30,31,32].

Therefore, food acculturation provides an important framework for understanding these changes, yet remains an understudied variable.

### 1.3. Food Acculturation and Chronic Disease Risks

Food acculturation—the process by which immigrants adopt the food and dietary practices of their new country [33], has been associated with chronic disease risk and overall deteriorating health [34,35,36]. In particular, unfamiliar food cultures and food purchasing environments, lack of knowledge on how to prepare and incorporate new foods in traditional diets, and perceptions towards food in new countries [33,37,38] limit immigrants’ food choices and access. As such, immigrants might be at higher risk of adopting diets that may have long-term negative health impacts, including diabetes. Food acculturation is not a simple shift from traditional diets to Western dietary patterns, rather, food acculturation is a multidimensional process shaped by cultural identity, social relationships, economic pressures, and structural constraints such as affordability, transportation, and access to culturally familiar foods [33,37]. These dietary transitions have important implications for long-term chronic disease risk.

In Minnesota, dietary changes among Somali and Ethiopian immigrant households occur within a broader set of structural and social determinants. Unfamiliar food environments, increased availability of processed foods, affordability concerns, transportation limitations, and reduced access to culturally familiar ingredients all influence food practices following migration [39,40,41]. At the same time, competing work and caregiving demands, language barriers, and challenges navigating new systems introduce persistent stressors that shape daily routines and health-related decision-making [5,41]. Within this context, food remains a central source of cultural identity and continuity following migration. However, maintaining traditional dietary practices can become increasingly difficult due to rising costs, limited accessibility and choices, and environmental constraints [42]. As a result, dietary change after migration is shaped not only by individual preferences but also by structural conditions and the lived realities of resettlement.

While studies in the U.S. and in other developed countries have investigated the changing diabetes status of African immigrants [21,22,23,25,30,32], very few in the U.S. have focused on immigrants from eastern African and the implications of food acculturation and diabetes risk factors [21,22,23,39,40,41]. Thus, the goal of this study is to provide a more contextualized and better understanding of the multilevel factors associated with food acculturation and diabetes-health related risk, specifically among East African immigrants, which may inform multilevel interventions and policies targeting immigrant health disparities. Additionally, limited U.S.-based research has examined how food acculturation is experienced within everyday life using qualitative approaches. Much of the existing literature relies on clinical indicators and fails to fully capture the cultural, household, and structural factors that influence dietary behaviors. This study addresses this gap by exploring the lived experiences of dietary change and perceptions of diabetes risk among East African immigrant women in Minnesota using qualitative data.

### 1.4. Conceptual Framework

This study is guided by the socioecological framework [43] to understand food acculturation among immigrants. Food acculturation is dynamic and multidimensional. Individuals do not move linearly from one end of the acculturation continuum to the other [33,37,44]. Rather, immigrants may find new ways to use traditional foods, consume new foods, exclude others, and/or incorporate them in their traditional diets [37,44]. Moreover, the process may be influenced by contextual factors at the individual, family/cultural, community, and structural levels. As such, the extent to which food acculturation influences diabetes-health related risks may depend on the path and the degree to which immigrants adopt the U.S. cultures, maintain their cultural practices, or both, and their environment. Figure 1 below is a visual representation of the food acculturation process and the multiple factors influencing it.

The socioecological framework was selected because it recognizes that dietary behaviors are shaped not only by individual knowledge and preferences, but also by broader family, social, environmental, and structural conditions.

## 2. Methods

### 2.1. Study Design

This qualitative pilot study explored multilevel facilitators and barriers to food and dietary acculturation among East African immigrant women living in the Twin Cities Metropolitan Area of Minnesota. Guided by the socioecological framework, the study examined how individual, interpersonal, community, cultural, and structural factors influenced food choices, dietary behaviors, food access, and perceptions of health following migration to the United States.

### 2.2. Study Setting and Participants

The study was conducted in the Twin Cities Metropolitan Area. It included six focus groups with a total of 36 East African immigrant women. Participants included Somali and Ethiopian women, including Oromo and Amharic-speaking participants. Women were recruited based on their role in household food preparation and food-related decision-making. As a result, the 36 women who participated in the study provided insight into their households’ dietary practices and food decision-making processes.

### 2.3. Recruitment

Participants were recruited using purposive and snowball sampling strategies through community-based outreach, word-of-mouth referrals, religious and cultural spaces, and community gathering locations serving East African immigrant communities. Recruitment approaches were designed to improve cultural accessibility, community trust, and participant engagement. Participants were included if (i) immigrant from Ethiopia or Somalia; (ii) women; (iii) adult—at least 18 years old; (iv) lived in Minneapolis–St. Paul metro area; (v) able and willing to sign the consent form—participants who showed up, but were unwilling to sign the consent form were excluded.

### 2.4. Data Collection

Data collection occurred between May and December 2025. Six focus groups were conducted using semi-structured interview questionnaires. Discussion groups lasted approximately 60 to 90 min and were conducted in participants’ preferred languages, including Somali, Amharic, and Oromo. Conducting focus groups in participants’ primary languages enhanced participant comfort, cultural expression, and richness of discussion regarding lived experiences. All discussions were audio-recorded with participant consent to preserve the accuracy and authenticity of participants’ narratives. All the focus groups were conducted by at least two members of the research team who speak the language of the participants. The PI was present and part of all the six focus groups. The PI also speaks both Somali and Oromo languages in which five of the focus groups were conducted, giving her the ability to cross check the transcription and translation. Additionally, all the research assistants spoke at least one language of the participants groups—two Somalis, an Oromo and Amharic speaker.

The focus group discussions explored food experiences after migration, food choices and preparation practices, dietary changes after migration, cultural and religious dietary practices. Barriers to access and availability, and factors that influence decision making in food choices, community support, experiences navigating the U.S. food environment, and diabetes-related knowledge, beliefs, attitudes, and prevention practices were discussed in-depth. Demographic information of participants was also collected.

### 2.5. Data Analysis

All recordings were directly translated into English shortly after each focus group discussion. The decision to translate directly into English was made to reduce the time it will take transcribing into the original language, then translating into English. The translation was first done by the two research assistants who attended the focus group. To strengthen credibility and accuracy, another research assistant and the PI listened to the audio and checked through the transcription to make sure nothing was left out. If there were any discrepancies, this was discussed during the weekly team meeting and the transcript corrected. After all the transcripts were cross-checked by at least two research team members, the PI invited certified medical interpreters from the community to double check the translated transcripts and make sure the transcripts could make sense to someone who was not part of the study.

A thematic analysis approach guided the data analysis following Braun and Clarke’s framework [45]. Research team members met in person and virtually to read and review transcripts. A codebook was then developed to capture recurring patterns, themes, and insights. This involved generating initial codes, defining them with clear examples to ensure consistency among coders, and refining the codebook throughout the analysis. Everyone then independently coded all responses to each question prompt, and at a subsequent meeting, compared their codes. Initial codes were manually developed from participants’ narratives. Initial coding agreement between the independent raters was 94% prior to resolving minor discrepancies through consensus discussions at subsequent meetings. Codes were organized into categories and broader themes using Excel spreadsheets to support theme development and organization. Themes and subthemes were refined through weekly research team meetings focused on identifying recurring ideas, phrases, experiences, and patterns related to food acculturation and health behaviors, reviewing interpretations, validating findings, and strengthening intercoder consistency. This collaborative process enhanced the trustworthiness and dependability of the findings while ensuring participants’ voices remained centered throughout the analysis. To further enhance the quality and transparency of reporting, the authors followed the Consolidated Criteria for Reporting Qualitative Research (COREQ) checklist [46]. Final themes were organized and mapped using the socioecological framework to capture interactions among individual, family, community, cultural, and structural influences on dietary acculturation among East African immigrant women. Data saturation was reached when no new themes or concepts emerged from the focus group discussions. Finally, select participant responses are included as quotes throughout this manuscript. Throughout the transcription and analysis process, the authors ensured that participants’ meanings, cultural context, and intended interpretations were preserved. This process was informed by Pshkin’s model of reflection [47] and the insider-outsider perspective [48].

Qualitative research requires acknowledgment of researchers’ influence. The research team consisted of two faculty members and research assistants from the social work and public health departments who had no close relationships with participants. Aware of our individual biases, we discussed during our initial meetings how our assumptions and beliefs can influence our interpretation of the data. Hence, during data analysis sessions, we made an effort to set aside our personal assumptions to avoid misinterpreting participants’ views. To ensure our interpretations reflect their responses, all the authors worked together to formulate the themes. We also made sure that the findings were grounded using participant quotes rather than the authors’ inference.

### 2.6. Demographic Characteristics of Participants

In addition to participation in the focus groups, participants completed a short anonymous demographic survey. As shown in Table 1 below.

A total of 36 East African immigrant women participated in the study. Participants represented Somali (N = 20), Amharic (N = 5), and Oromo-speaking (N = 11) communities. Length of residence which is an important factor in HIE ranged from recent arrivals (≤4 years) to long-term residents (>10 years). On average, participants have lived in the U.S. for 11.6 years with a wide variation ranging from six months to over 30 years. Over a third (13) of them have been in the U.S. for less than five years and over half have been in the U.S. for more than 11 years. The mean age is 47 (15.05) years with a large variation ranging from 22 to 70 years old. The average household size is 5.2 (2.54), the average number of children per household is 2.8 (2.2) again with a wide variation ranging from no child under 18 to seven children under 18.

## 3. Results

Thematic analysis identified two overarching themes related to food and dietary acculturation among East African immigrants in the Twin Cities, Minnesota: (1) individual and family-level factors and (2) community and structural factors. Findings reflected the complex ways migration influenced food choices, dietary practices, physical activity, and perceptions of diabetes risk. The comparison across focus groups revealed recurring meanings alongside differences within and between discussions. Health-related statements reflect participants’ perceptions and reported experiences. Table 2 (Thematic Summary Matrix) summarizes these patterns and their variation; the supplementary evidence matrix reports their distribution across the coded material. Quotations are labeled by focus group (FG) and de-identified participants (P).

Table 2 below presents a thematic summary matrix. The first five rows are individual and family-level factors (Theme 1); the final three are community and structural factors (Theme 2). Component names are shortened for display. Group references locate evidence, not consensus or frequency. The supplementary matrix contains the complete group-by-component comparison.

### 3.1. Theme 1: Individual and Family-Level Factors

We identified several individual and family-level factors that may influence food and dietary acculturation among participants, and these are discussed below.

#### 3.1.1. Individual Factors

##### Emotional Attachment to Ethnic Foods

Participants expressed strong emotional, cultural, and health-related attachments to traditional foods. Traditional foods were perceived by most participants as healthier, more natural, and more satisfying than American foods. Many participants associated these foods with cultural identity, familiarity, and a connection to home. Maintaining traditional dietary practices was viewed as an important way of preserving culture while supporting personal preferences and family health.

One woman described, *“We are always looking for the foods we grew up with”* (FG1, P3). In FG3, difficulty adapting to food was connected with longing for home: “*Yes, the taste has changed. I sometimes say, … I could not adapt to the food at first*” (FG3, P19). An FG4 participant explained the emotional significance directly: *“When I can’t eat familiar foods, I feel uncomfortable”* (FG4, P24). These accounts place familiarity in relation to comfort, preferred tastes, and a connection to home.

Familiarity also shaped which foods participants could accept. A woman in FG2 stated, *“I only know meat, rice, basto, and anjeelo (Somali crêpes). That’s it. Something I don’t know can’t become food for me”* (FG2, P10). Returning to known foods was one response to difficulty adapting: *“Sometimes, when we struggle to adapt, we go back to foods we know, like beans”* (FG3, P15). An FG4 participant also reflected*, “At first, it was difficult to adjust to the taste of the food, but eventually I returned to eating familiar Ethiopian foods”* (FG4, P22). Thus, continuity could involve returning to familiar dishes as well as retaining them.

The connection between cultural attachment, taste preference and perceived health was also shared. One woman explained, “*We choose these foods because we grew up with the taste, enjoy them, and believe they are healthy, gluten-free, and helpful for managing weight”* (FG6, P32). Another supported similar view stating, *“We believe our cultural foods are much healthier and the best choice for maintaining good health”* (FG6, P33). However, there was also variation in the perceived healthy choices connected to traditional foods and plans to modify them. In FG1, a participant voiced concern about meat and cooking oils: *“For example, goat meat and cooking oils are not good*” (FG1, P4). Another described how attachment to a familiar dish coexisted with a shift to an ingredient: “*As Somalis, we love our traditional crepes, but it isn’t healthy if it’s made with sugar. I’ve reduced the sugar*” (FG1, P2). These statements suggest that valuing cultural foods does not entail treating every ingredient or preparation pattern as healthy. While participants often contrasted traditional foods with American foods, which were commonly perceived as highly processed, artificial, and less nutritious, their narratives also illustrate that maintaining cultural practices can involve uncertainty, questioning ingredients, changing preparation recipes, and combining other dishes.

##### Sustainability of Dietary Changes

Although participants demonstrated awareness of healthy eating recommendations and perceived diabetes-related risks, maintaining long-term dietary changes was described as difficult as it needs specific adjustments and persistent efforts. Changes in family dynamics after migration such as family responsibilities, childcare obligations, work demands, time constraints, and competing priorities frequently limited participants’ ability to sustain dietary modifications. Responding to a discussion of dietary recommendations, a woman commented, *“That’s hard to do, especially when you’re cooking for your family and feeding children. You end up giving in to temptation” (FG1, P6).* A participant in FG3 expressed similar concern *“Even when we try to follow a diet to prevent diabetes, it is difficult for families to maintain”* (FG3, P19). Another participant expressed a perceived pattern in her community: *“We follow instructions for a few days, but then we go back to our old ways”* (FG1, P1). FG2 narratives correlated difficulty persisting changes with unfamiliar foods and a demand for practical guidance. One woman linked a period of dietary change to her blood sugar readings: *“If I stop drinking tea and I stop eating rice for a while, there will be many days where my sugar will be good”* (FG2, P8). She then clarified, *“But the question is I can’t continue it because these are foods foreign to me*” (FG2, P8). The same participant described the importance of guidance: *“If you don’t know and you’re not given the right answers to change what you eat into something better, it’s very hard” (FG2, P8).* A woman in FG5 likewise stated, *“Even when we try to follow a diet to prevent diabetes, it is difficult for families to maintain because we are used to eating many different foods and find it challenging to reduce or consistently follow a plan*” (FG5, P1).

Participants described balancing health recommendations with the realities of daily life in a new country, including limited time for meal preparation and the challenges of meeting the dietary preferences of multiple family members. Despite these barriers, many reported actively attempting to improve their diets by reducing sugar intake, modifying recipes, increasing vegetable consumption, and monitoring foods that affected blood sugar levels. As one participant explained, “*We are already working on improving our diets, and we believe that if we monitor what we eat and maintain the quality of our cultural foods, it will help protect our health”*. (FG6 P34). An FG2 participant similarly expressed initial resistance followed by change, as she recalled, *“You know my son, he kept telling me to stop adding honey to my milk. I said I won’t stop. I went to my doctor, and I told her I use honey in a lot of my things”* (FG2, P9). Later, she described changing her drinks and how she felt: *“I stopped the honey too. Now I drink milk on its own. I used to crave sweetness and sweet taste, but now I’m not like that anymore. I even feel healthier”* (FG2, P9). FG3 also discussed diagnosis-related changes and educator guidance. One participant described, *“I have diabetes, so I cook with whole wheat. I make whole wheat crepes and whole wheat meals*. *I also cook vegetables for lunch, like beans and carrots” (FG3, P14).* Further, she stated, “*A diabetes educator helped me. She explained portion sizes and what to eat: beans, watermelon, mango, and low-carb foods*” (FG3, P14). These statements identify reported dietary adjustments in the context of managing diagnosed diabetes and accepting practical advice. The narratives in FG 4 and FG 6 also reveal how participants conceptualized and demonstrated dietary continuity across time. For example, one noted, *“I want to improve my diet and exercise, and I am willing to change, but sometimes food options are limited*” (FG4, P22). Another described attempts alongside concerns about appropriate options: *“I try to reduce carbohydrates, exercise, and check my sugar, but it is hard because many foods contain sugar” (FG4, P23).* Another framed continuous effort through a belief in their protective value: *“We are already working on improving our diets, and we believe that if we monitor what we eat and maintain the quality of our cultural foods, it will help protect our health”* (FG6, P35). These statements depicted intentions and efforts to sustain changes even though practically challenging.

##### Stress

Stress emerged as an important factor influencing dietary behaviors and perceived health risks. Participants described financial pressures, caregiving responsibilities, migration-related challenges, and daily demands as contributing to emotional stress and difficulty maintaining healthy lifestyles. In FG1, a participant related pressure to school routines: *“Here we have fixed schedules. If your kid is late to school, it becomes a problem. The school system is different. There is a lot of stress”* (FG1, P2). Describing responsibilities at home, she added, *“You come home tired, have to cook, help with homework, give kids attention, and then go to bed*” (FG1, P2). Her narrative connects experienced stress with coordinating school, cooking, and children’s needs. Similarly, participants in other groups tied the broader demands of family life in the U.S. with understanding of stress and health: *“These lifestyle changes, combined with larger family responsibilities, contribute to higher levels of stress and increased health risks*” (FG6, P32). Another stated, *“I believe that stress and overthinking can raise blood sugar levels and contribute to diabetes”* (FG5, P4), another one expressed similar sentiment: *“We simply don’t feel the same desire to eat in the U.S. This may be due to stress or overthinking, or because the foods we grew up eating were completely organic, something we cannot be sure of here*” (FG5, P5). These statements suggest stress both as an experienced burden and as one part of participants’ explanations of food and health decisions. Financial demands were also shared as extra burden and stress impacting their lives. In FG6, a participant described financial demands as daily pressure: *“The stress of bills, taxes, and basic needs also adds pressure”* (FG6, P32). Participants linked stress to unhealthy eating patterns, reduced motivation for physical activity, and difficulties sustaining recommended lifestyle changes. Stress was therefore viewed as both a direct and indirect contributor to chronic disease risk.

#### 3.1.2. Family Factors

##### Intergenerational Food Preferences

Participants described substantial differences between adults and children in dietary adaptation and food preferences after migration. While adults generally preferred traditional foods, children often adapted more quickly to American foods, including fast food, takeout, and convenience foods. These differences may have sometimes created tension within households and increased parents’ burden of meal preparation and food budgets.

One woman depicted the contrast as *“We are two countries that live together. People who grow up here and are people of this country they’re different. When you buy pizza, the kids are happy. But when you give them rice, you have to add ketchup for them”* (FG2, P8). In FG5, one participant described: *“In general, the children prefer American foods, while we adults favor our cultural dishes prepared at home”* (FG5, P3). Differences were also viewed among children in one household: *“I have both younger and older kids. I notice the older ones prefer junk food. Some eat what I make, but others ask for fries or chicken nuggets. They are all different”* (FG1, P2). These statements uncover variation both between generations and within the same generation.

These intergenerational differences had practical consequences as expressed in the following participants statements: *“Our children also struggle to accept the taste of our cultural foods, so we often must prepare separate meals for them, which adds extra expense, and time”* (FG6, P32). Another woman associated changing preferences with schooling: *“But once they start school and get used to American foods, it becomes difficult to include traditional dishes in the family menu”* (FG6, P33). A woman in FG3 explained, *“We try to make crepes every day in bulk, but no one eats it. It sits in the fridge until we throw it out. The kids mostly want juice, chips, chicken, or packaged food”* (FG3, P19). An FG1 participant stated the household division and the work it involved: *“My house feels like two countries and different food cultures during meal time. Kids prefer and eat American foods, while we parents are stuck with our traditional food that we used to eat back home. It becomes exhausting to cook food*” (FG1, P6). These narratives associate children’s tastes with extra work, higher costs, and reduced space for familiar dishes in family meals.

Exposure to American foods through schools, peers, and the broader food environment appeared to accelerate dietary acculturation among children. Despite these challenges, many participants continued preparing traditional meals and encouraging cultural food practices within their households, reflecting ongoing efforts to preserve cultural dietary traditions across generations. Some participants described adapting American foods to fit their household preferences by preparing them at home alongside traditional meals. As one participant in FG5 shared, “*Sometimes, when we want variety, we intentionally decide to eat American food. However, what I dislike about American food is the way ingredients are combined. Even when we try to prepare these dishes at home…*” (FG5, P25). Another participant explained, “*We mainly eat our traditional foods, although we occasionally combine them with dishes from other cultures*” (FG5, P26). These accounts suggest that dietary acculturation also involved modifying and integrating American foods rather than fully adopting American dietary patterns.

##### Changing Family Roles, Schedules, and Mealtime Disruptions

Migration altered family dynamics, routines, meal patterns, and food preparation practices. Participants described how work schedules, school commitments, transportation demands, and competing responsibilities reduced opportunities for shared family meals and changed traditional approaches to cooking and eating. One participant connected work and school with limited cooking time: *“The lifestyle here doesn’t allow for daily cooking. People go to work or school. Time is limited”* (FG1, P3). Another depicted the school-day routine: *“Here, mornings are rushed. You are dropping kids off at school”* (FG1, P2). Responding to a researcher’s comparison of shared meals, she also confirmed, *“Yes, back home we cooked and ate at the same time together”* (FG1, P2). Another one supported, “*Back home, the whole family would eat together. Here, even if you cook, people are at school, or work”* (FG1, P3)

Participants also described changes in food purchasing and storage practices. A woman in FG5 contrasted both practices in the same account: *“Back home, we shopped for fresh food every day, but in the U.S., shopping only happens occasionally. Here, people cook and store food in the refrigerator for several days, whereas back home, the food is cooked fresh daily and does not develop strong smells”* (FG5, P5). Preparation time was also clear: *“One challenge, however, is that preparing these foods is time-consuming”* (FG6, P31). Employment could also affect the food provision. An FG2 participant described packing prepared meals for a truck driver: *“Some days I make a lot of rice. He drives a truck, so I put it in containers”* (FG2, P7). These examples connect food choices to the effort and coordination needed to provide them.

In addition, convenience carried mixed meanings. One participant described missing daily access to fresh food while welcoming less preparation: *“But I miss going out and getting fresh food every day. I liked that part. At the same time, not having to cook crepes every day is also a relief”* (FG1, P1). These findings suggest that dietary acculturation involves not only changes in food choices, but also changes in family dynamics, household responsibilities, meal preparation practices, and family interactions surrounding food.

### 3.2. Theme 2: Community and Structural Factors

#### 3.2.1. Availability and Affordability

Participants consistently identified the availability and affordability of culturally familiar foods as challenges after migration. Traditional foods and ingredients were often more expensive than mainstream foods and were not always available in local grocery stores.

Cost as a barrier was shared across groups. One participant stated, “*Traditional foods are harder to find, and everything is more expensive*”. (FG1, P2). Another participant explained, “*The cost, especially since teff* (gluten-free, nutrient-dense flour native to Ethiopia*) flour is expensive and the prices are not the same as back home*” (FG6, P34). In the same group another participant also shared *“American stores do not carry many of the items we need”*. (FG6, P31). In FG4 cost was also identified as a barrier, *“Meat is available, but it is expensive, and I don’t know how fresh it is”* (FG4, P24). Expenses also arose from organizing family meals. In FG6, a participant related children’s preferences to additional preparation: *“Our children also struggle to accept the taste of our cultural foods, so we often must prepare separate meals for them, which adds extra expense, and time”* (FG6, P32). Affordability, therefore, involved both ingredient prices and the demands of preparing acceptable meals for different family members.

Perceived quality was also regarded within a limited food budget: *“So the high-quality groceries are more money that’s the problem. We don’t have the budget*” (FG2, P13). Providing different ingredients could require several shopping trips. In FG5, a participant described, *“For example, if I go to Walmart, I can buy basics like rice and oil, but it is hard to find many cultural ingredients or traditional food items. Because of this, I usually must visit two or three different stores to get everything I need to cook our cultural foods”* (FG5, P5). An FG6 participant similarly explained, *“Availability and access play a big role in our shopping habits, we often need to go to cultural grocery stores because American stores do not carry many of the items we need”* (FG6, P32). The presence of a store, therefore, did not necessarily confirm that it offered the products a household wanted at an acceptable price or quality.

Substitution provided another response to limited options. One FG4 participant said, “*When cultural foods are limited, I eat rice”* (FG4, P24). Another explained, *“I eat pasta, pizza, and white bread when I don’t have access to cultural foods. These foods are quick, easy, and save time”* (FG4, P23). In FG6, choices also reflected price and family preferences: *“Our children’s preferences and the lower cost of certain American foods influence these choices”* (FG6, P32). Availability and affordability shaped household trade-offs in diverse ways, including convenience, taste, and family acceptance, rather than producing a single common pattern of meal choice.

#### 3.2.2. Access as a Barrier

Transportation and geographic barriers also influenced access to preferred ethnic grocery stores and culturally familiar foods. Participants described long travel distances, sometimes limited transportation options, and reliance on family members or friends to obtain traditional ingredients. This was particularly significant for newcomers. Reflecting a change in local provision, an FG1 participant said, *“Yes, it was harder before. Now we have Somali grocery stores and Halal markets*” (FG1, P3). Another distinguished earlier shopping with her current reliance on transport: “*Back in South Africa, I could walk to get groceries. Here, I need a car and someone to drive me”* (FG1, P6). Local availability and physical access to a shop, therefore, formed distinct dimensions of food access.

Distance could influence shopping across different kinds of stores. One participant explained, *“Transportation is a major challenge, and I often must rely on others to help me get these foods”* (FG4, P22). Similar sentiments were shared in FG5, “*However, buying certain traditional items, such as injera, is difficult for me because I do not drive”* (FG5, P28). Another participant stated, *“Living in the suburbs adds to the difficulty because cultural stores are far away*”. (FG6, P32). She continues “*We often have to drive from the suburbs to Minneapolis just to buy certain ingredients*”. (FG6, P32). Exploring shops was one practical response: *“To find cultural groceries, we usually search for stores online”* (FG6, P34). Receiving preferred foods could therefore require planning, even where shops were available in the broader area.

Knowing how to navigate available shops was also discussed. Alongside her explanation of accessible halal stores, an FG2 participant referred to unfamiliar alternatives as “*The grocery aisles that we don’t understand”* (FG2, P8). She also expressed how constrained alternatives could make nearby stores the practical choice: *“If they can’t go somewhere else, they have to shop at these Halal stores”* (FG2, P8). Familiarity with the neighborhood and access to other shops thus influenced participants’ choice of where to shop.

Access also involved recognizing products that met participants’ requirements. An FG1 participant explained, “*The most surprising thing is checking if everything is halal. Even with yogurt and cereal, I have to double-check the ingredients. I am constantly checking for gelatin”* (FG1, P6). An FG3 participant linked this task with reading skills: *“If you can’t read labels, it’s hard. Young people can read labels that help them”* (FG3, P15). These statements broaden access to include not only physically reaching a store, but also understanding and evaluating the products in the market.

#### 3.2.3. Migration-Related Stress, Sedentary Lifestyle, and Exposure to Highly Processed and Sugary Foods

Participants described substantial lifestyle changes following migration that might have affected dietary behaviors and health. Many reported becoming less physically active and more exposed to processed, sugary, and convenience foods than in their countries of origin. Across the groups participants shared how physical activities have decreased post-migration and car dependency increased, and the implication for their health as evidenced in the following participants statements: *“Back in South Africa, I could walk to get groceries. Here, I need a car and someone to drive me*” (FG1, P6). An FG2 participant says *“The exercise itself is work, but we had the exercise, we had the food that helped us, we would walk often. We had the fresh nutritious foods. But here we use our cars all the time”* (FG2, P9). In FG5, one recalled, *“Back home, daily life involved more movement and small activities that kept the body active”* (FG5, P2). These suggest daily chores in shopping, work, and household routines as helpful physical activities rather than only in planned exercise. Further examples connecting daily tasks to physical activities include: *“Back home, we had plenty of physical activity as part of daily life. Here, life is more sedentary, food is easier to prepare, and we spend much more time sitting”* (FG6, P32). Another adds in the same group *“When we were back home, we stayed physically active by walking to shop, to fetch water, and during other errands, and this helps us regulate and maintain our overall health”* (FG6, P34).

Changes in food and eating routines were similarly shared and related to health outcomes. An FG1 participant connected snacks to school and work in the U.S: *“We go to school, we take snacks. At work, we take snacks”* (FG1, P2). Another described snacking as a familiar practice: *“Here, we are used to having snacks all the time”* (FG1, P3). Noting a return visit to Somalia, a participant distinguished the meal pattern she encountered: *“There, people eat breakfast and wait until lunch. There are no juice or snacks in between”* (FG1, P2). These discussions also included access to easy fast foods. In FG6, a participant shared: *“I was 16 years old when I first moved to the U.S., I used to eat at McDonald’s very often. Over time, I realized the way the food was cooked, the oil, the pans, and the overall quality, was unhealthy. So, I stopped eating it and returned to preparing cultural foods. I even started making injera myself, something I never did back home, and I felt much better afterward”* (FG6, P33). An FG4 participant linked food, movement, and uncertainty about food quality in her explanation: *“Food in the U.S. feels heavier than food in Ethiopia. I used to eat three or four meals a day, but now I eat only two because the food feels heavy. I think this is because I move less or because the food may not be organic”* (FG4, P23). An FG6 participant associated blood-sugar changes with shifts in her diet: “*It took me a long time to adjust to the taste of American food. When I started eating more of it, I noticed my blood sugar increasing, and I was diagnosed with diabetes. However, when I went back to eating more of my cultural foods, my blood sugar became more stable”* (FG6, P35)

Migration-related stress further contributed to perceived health risks. Participants linked financial pressures, caregiving responsibilities, adaptation to a new environment, and changing lifestyles with unhealthy eating patterns and increased perceived risk of diabetes and other chronic diseases. Participants also expressed uncertainty about the causes of nutrient deficiencies and chronic disease, despite feeling they consumed adequate amounts of food. Several described receiving information about iron deficiency, high cholesterol, or diabetes risk from healthcare providers while struggling to understand how these conditions developed when they believed they were eating sufficient amounts of food. Some questioned why health problems occurred despite consuming traditional foods that they considered healthy and nutritious. These concerns highlighted perceived gaps in nutrition knowledge, health communication, and understanding of chronic disease prevention.

Overall, findings suggest that dietary acculturation among East African immigrants is shaped by complex interactions among individual, family, community, and structural factors. While participants faced barriers related to affordability, transportation, changing family dynamics, stress, and exposure to highly processed foods, they also demonstrated resilience through the continued consumption of traditional foods, preservation of cultural food practices, adaptation of recipes, utilization of ethnic food stores, and reliance on community support networks.

## 4. Discussion

The goal of this qualitative study is to explore how East African immigrants in the Twin Cities, Minnesota perceive and describe the multi-level protective and risk factors of diabetes associated with food and dietary acculturation. Participants’ narratives, interpreted through a socioecological framework, describe food acculturation as a multidimensional experience associated with factors across individual, family, community, and structural domains. In these accounts, a strong cultural attachment to traditional foods and practices coexists with adaptations to unfamiliar food environments, shifting family dynamics, and structural barriers such as cost and transportation. These contextual narratives are supported by previous evidence showing that dietary acculturation depends on a range of multi-level factors, including ethnic and non-ethnic cultural ties and food-environmental factors such as food costs, access, and quality [49]. In addition to the individual and structural-level barriers, the new environment also comes with increased exposure and easy access to highly processed foods, a finding also reported in this study [50].

At the individual level, participants expressed strong preferences for their traditional and familiar foods, which they consistently described as healthier, tastier and more natural. Taste and familiarity with the food and ingredients played a key role in food choices, corroborating a previous study that found that taste was a very or extremely important factor in food choice for 82% of participants [51]. Additionally, participants perceived traditional/ethnic foods as healthier because they know how and where these foods are produced, a perspective tied to both their choice to maintain traditional dietary practices and their views on managing diabetes-related health risks. Furthermore, to the participants, ethnic food is not only a source of nourishment, but also a way of cultural preservation, social cohesion and emotional connection to their homelands. It serves as a continuity of identity and belonging after migration. However, cultural continuity could also mean learning new cooking skills after migration and returning to familiar foods after experimenting with fast foods. It is interesting to note that previous acculturation studies among second generation U.S. college students found a similar connection to cultural foods as it served as cultural anchors that preserved participants’ cultural identity and protected against feelings of cultural loss [52].

Another important finding at the individual level is that while participants had some awareness of correlations between diet and health, building a sustainable dietary change or behavior was a challenge. Participants described putting efforts to change their dietary patterns mostly after a doctor’s recommendations due to perceived health risks such as pre-diabetes or diabetes diagnosis. They consistently expressed difficulty maintaining long-term dietary change, due to work and caregiving responsibilities, high stress levels, and affordability issues.

Faith and religious guidelines regarding food also emerged as an influential factor on where participants shop for groceries and the foods they consume, specifically meat. Most of the participants mentioned they only eat “halal” meat, which is prescribed by their belief system. Even though meat is more expensive at the ethnic grocery stores compared to the conventional American grocery stores, participants shared buying meat products strictly from ethnic stores or locations where they can verify the product as “halal”. The influence of faith, religion and culture in food choices and decisions is well established in the literature in a variety of settings [42,53,54].

Family level factors also contribute to the complexity of food acculturation. Participants shared that changes in family roles and family dynamics have disrupted family meal practice after migration. Shared family meal times became difficult due to work and school schedules, and other competing responsibilities, especially for women. Participants have shared using practical adaptation to reduce the impact of mealtime disruptions such as cooking together and shared decisions. Family mealtime disruptions may have implications for social support, family cohesion and long-term health behaviors as prior research has demonstrated that regular family meals are associated with healthier dietary behaviors, improved psychosocial outcomes, and reduced chronic disease risk [55,56].

Intergenerational differences in food acculturation and dietary habit emerged as a key family level factor in this study. Participants consistently perceived children as adapting more quickly to American foods and developing a stronger preference for fast foods. This might have influenced households’ dietary patterns and meal routines, while in some instances may also be contributing to tensions within families. Participants shared that they prepare separate meals for children and adults and find it difficult to maintain the traditional food practices within the family. These findings suggest food acculturation and dietary patterns after migration extend beyond individual behavior and preferences. It involves negotiation of identity, belonging and adaptation across generations. Consistent with previous studies, exposure to school food and peer influences accelerates food acculturation among children much faster than parents and adult family members [57].

The study findings also reveal the influences of both community and structural barriers towards food acculturation and dietary patterns. Availability of stores that carry ethnic foods, higher cost of those foods and ingredients, and transportation as ethnic stores are limited, all emerged as barriers to keeping traditional diets that participants valued as a healthier food option. Transportation issues are a particularly significant barrier for newcomers. The findings also suggest unfamiliarity with the shopping environment and the U.S. food system such as unfamiliar products, understanding labels, language comfort and unfamiliar grocery negotiations as barriers in the U.S. food environment.

In addition to food and dietary changes, broader structural conditions, including sedentary lifestyles, migration-related stress, and increased exposure to processed, sugary and convenience foods, were perceived as contributing to deteriorating overall health and elevated diabetes risks after migration. Participants described walking and physical activity as part of their daily lifestyle to meet their family needs and not a leisurely activity in their home country, whereas in the United States, they cannot just walk for groceries due to several factors including proximity, weather and a culture of car dependence. These findings align with previous studies that have documented increasing prevalence of diabetes and diabetes risk factors after migration among Somalis, Ethiopians and other African immigrants [23,28]. Participants’ descriptions of sedentary lifestyles after migration also support earlier studies indicating that migration to high-income countries often reduces routine physical activity that require walking and manual daily activities that are part of their lives prior to migration [58,59].

Participants viewed community support systems as a helpful presence in navigating these barriers, describing them as a valued resource in making their food choices and decisions. The findings suggest newcomers receive strong social and community support as they navigate unfamiliar food systems, which helped them maintain cultural food practices after migration. Community members provided assistance related to transportation, interpretation, grocery shopping guidance, and navigating unfamiliar grocery environments.

Although the current study is exploratory, the findings expand the existing literature in important ways. There is limited research examining the role of food acculturation on health outcomes among East African immigrants in the United States, particularly women who are primarily responsible for household food preparation and dietary decisions. By centering the lived experiences of immigrant women, the study provides nuanced insight into how food acculturation is experienced and negotiated within daily life. The findings also contribute to our understanding of food acculturation as a complex, multidirectional and ongoing process rather than a linear process from traditional to Western dietary practices. This is supported by the work of [33] multidimensional acculturation framework. The study findings reveal that participants maintained aspects of traditional diets while adapting to new food environments and negotiating competing household demands.

While previous research suggest that immigrant children in the United States adapt faster than their parents in all aspects of life including food, particularly through exposure to school food environments and friends [57,60]. Our study builds on this perspective by highlighting how children’s food preferences and adaptations are associated with shifts in household dietary practices and accelerated food acculturation for immigrant households. Participants described these changes not simply as dietary shifts, but as broader changes in family routines, cultural practices, and social relationships. Furthermore, our findings reinforce existing evidence that structural and environmental factors are strongly associated with immigrant health behaviors. Similar to prior studies, participants identified cost, transportation, neighborhood food environments, and work demands as key perceived influences on their food access and dietary behaviors. These findings support socioecological models of health behavior, which recognize that individual choices are shaped by broader social and structural contexts.

### 4.1. Implications for Practice and Research

The study findings have important implications for practitioners, including healthcare providers, public health practitioners, and community organizations working with East African immigrant populations. At the individual level, diabetes and nutrition interventions should be culturally responsive and grounded in participants’ lived experiences. It is important for practitioners to recognize the cultural significance of traditional foods and recommend interventions that build upon existing strengths within traditional diets while collaboratively identifying culturally appropriate modifications necessary for health benefits.

At the family level, family-centered approaches may be important for addressing intergenerational dietary changes. Programs targeting both parents and children may help strengthen healthy family meal practices, support cultural food traditions, and help reduce tensions surrounding dietary preferences within households. Schools and youth programs may play an important role in promoting culturally inclusive nutrition education and healthier food environments for immigrant children. It is encouraging that some of these practices are already happening, for example, within the Twin Cities Metro area, school districts have incorporated ethnic foods from the different communities represented in the region [61,62].

It is equally important to address community and structural barriers that limit healthy food access. Designing programs that improve access to traditional foods, transportation assistance and culturally appropriate grocery navigation education for newcomers may help reduce some of the barriers participants identified and help maintain healthy dietary practices. Specifically, preferred-language product guidance, partnerships with cultural retailers, connections to foods assistance, affordability, and access for families with fewer community connections. Community health worker models and peer-led programs may also be helpful in reducing the structural barriers given participants’ emphasis on community support and shared cultural knowledge. This recommendation is also corroborated by previous studies [63].

### 4.2. Study Limitations

While this study provides important contextual insight into food acculturation and perceived diabetes-related risk factors among an understudied immigrant population, the discussed limitation should be considered when interpreting these results. Our sample size is a relatively small group of Somali and Ethiopian women from the Twin Cities Metropolitan Area, which may limit generalization of our findings to all immigrants, cultural context or those in vastly different geographic settings. The study relied on non-probability convenience and snowball sampling to recruit participants. Recruiting via faith-based organizations (e.g., local mosques) and cultural centers likely overrepresented individuals who are actively engaged in community life. This approach introduces potential selection bias, as individuals who agreed to participate may have had a stronger personal interest in diabetes, greater exposure to informal health information, or stronger community ties than non-participants. Additionally, data collection depended entirely on self-reported qualitative interviews, making findings susceptible to social desirability bias. Participants may have overreported adherence to healthy eating or traditional food practices and underreported consumption of processed foods or sedentary habits to align with perceived expectations of the group or the researcher. Although the focus groups were conducted by bilingual research assistants and the PI, who is multilingual, the process of translating concepts from native East African languages (e.g., Somali, Oromo, Amharic) into English introduces potential translation bias, since some subtle meanings, cultural expressions, or feelings about health and food may have been altered or lost. This study exclusively focused on East African immigrant women. While women often serve as the primary food gatekeepers within households, the exclusion of men limits the transferability of our findings across the broader East African community. Men may encounter distinct gendered acculturation stressors, workplace dietary constraints, and health-seeking behaviors that were not captured in this study. While participants shared a common identity as East African immigrants, significant heterogeneity existed in the sample across country of origin, ethnic background, age, U.S. length of residence, English proficiency, and socioeconomic status. Grouping these diverse lived experiences under a single analytical umbrella may mask important subgroup differences in dietary acculturation and perceived health outcomes. Finally, our study did not collect objective dietary data (e.g., 24 h dietary recalls or food frequency questionnaires) or physiological biomarkers (e.g., HbA1c or fasting blood glucose). Hence, reported dietary patterns cannot be used to infer causation, objective dietary intake, or clinical cardiometabolic risk. Nevertheless, these qualitative findings provide valuable insights into participants’ lived experiences, dietary changes after migration, and perceptions of diabetes in relation to diet, offering important evidence to inform the development of culturally tailored diabetes prevention and management interventions, health education programs, and community-based strategies for East African immigrant populations. Furthermore, these findings provide a strong foundation for future mixed-methods and longitudinal research that integrates qualitative findings with objective clinical and behavioral measures to understand perceived diabetes risk better, evaluate intervention effectiveness, and strengthen culturally responsive diabetes prevention and management efforts.

## 5. Conclusions

This study explored East African immigrants’ women perceptions of the multilevel factors influencing food acculturation and their perceived relationship to diabetes. The findings demonstrate that food and dietary acculturation is not a linear process where immigrants shift from traditional to western based diet. Rather, it is shaped by complex interactions across individual, family, community, and structural factors. Participants expressed strong desires to maintain traditional dietary practices, which they perceive as healthier and a protective factor, but faced substantial barriers related to affordability, accessibility, transportation, changing family dynamics, stress, and exposure to processed food environments after migration.

As discussed in the implications, these findings suggest the need for public health approaches that go beyond individual-level interventions and address broader social and environmental factors that influence food acculturation. Efforts that improve access to culturally appropriate foods, strengthen community-based nutrition education, and center on culturally responsive, community-informed strategies may better support sustainable dietary change and perceived diabetes risks among East African immigrants.

## Figures and Tables

**Figure 1:**
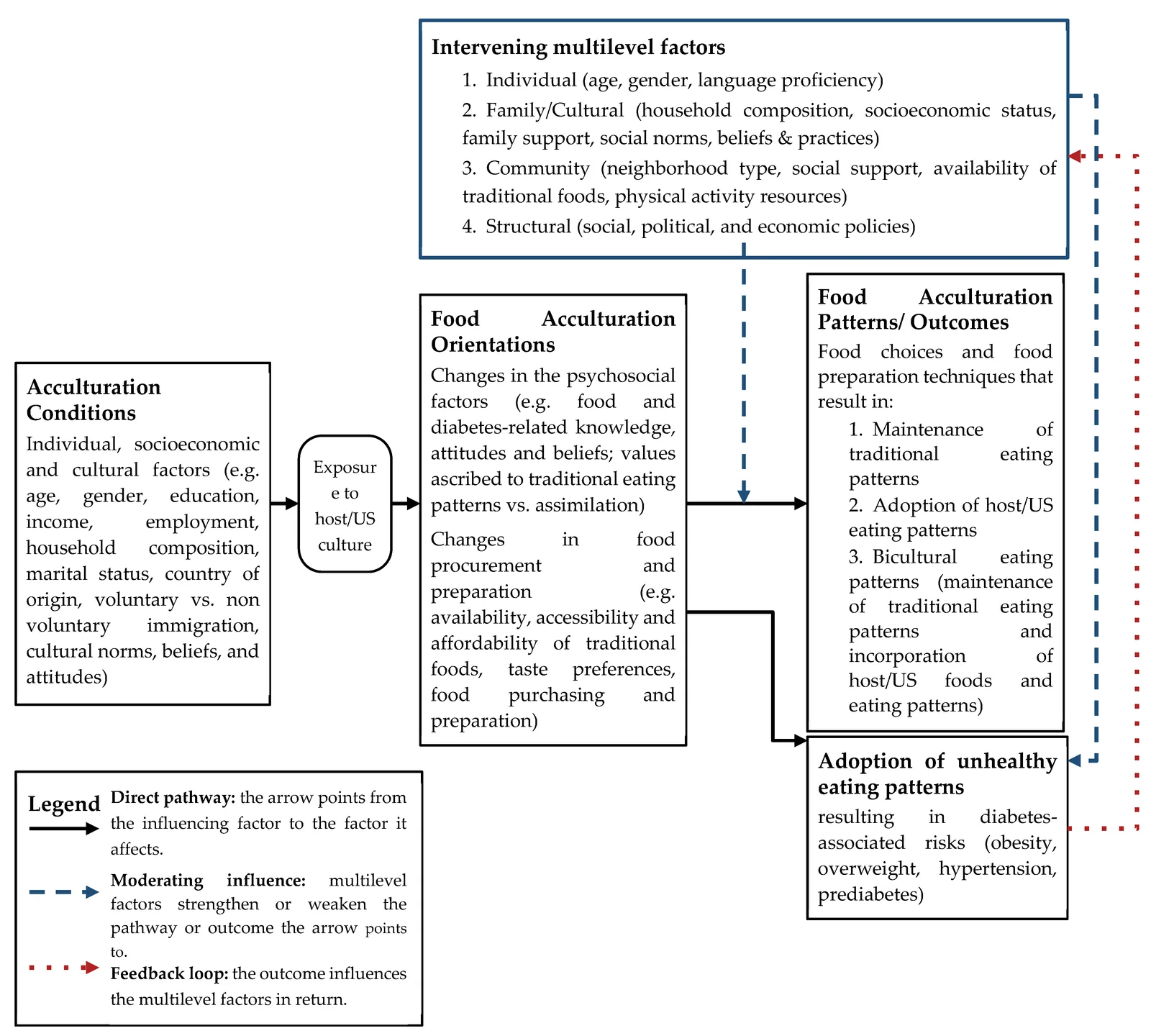
Conceptual model for food acculturation and diabetes-associated risks among immigrants.

**Table 1:** Participants Demographics.

Variable	N (%)/Mean (STD)
Age (mean)	47.05 (15.05)
No. of years in the U.S. (mean)	11.67 (9.34)
Average House Hold Size	5.2 (2.54)
No. of children under 18 (mean)	2.8 (2.22)
Marital Status Married Widowed Divorced/separate Never married Not answered	20 (55.5%) 9 (25%) 3 (8.33%) 3 (8.33%) 1 (2.8%)
Level of Education College and some college High School Middle and Elementary No school No response	5 (13.9%) 12 (33.3%) 10 (27.8%) 8 (22.2%) 1 (2.8%)
Employment Status Fulltime Part time Stay at home mom Retired Not employed Student	8 (22.22%) 8 (22.22%) 9 (25%) 6 (16.66%) 3 (8.33%) 2 (5.55%)
Total participants	36

**Table 2:** Thematic Summary Matrix (themes, recurring meanings, and qualifying accounts).

Themes	Recurring Meaning and Reported Practices	Variation and Source Illustration
Individual emotional attachment	Familiar foods offered continuity, comfort and cooking knowledge across FG1–FG6. Reported practices included retaining cultural meals, modifying ingredients and combining dishes.	Health beliefs varied. FG1 questioned ingredients; FG2 included nutritional uncertainty. “We’re always looking for the foods we grew up with” (FG1, P3).
Individual sustainability of changes	Difficulty fitting advice into familiar tastes and family routines coexisted with changes supported by educators or relatives. FG4 provided limited depth on duration.	Stopping a diet did not mean stopping change: “I changed my eating habits” (FG1, P2). FG2 included resistance followed by reported change; intentions did not establish maintenance.
Individual stress	FG1, FG5 and FG6 explicitly described strain or stress beliefs. FG2 financial context, FG3 diagnosis-related emotion and FG4 shopping unease provided narrower coverage.	“The stress of bills, taxes, and basic needs also adds pressure” (FG6, P32). FG1 also included relief from earlier cooking and shopping demands.
Family intergenerational preferences	Preference differences could add meals, work and cost. Direct comparisons occurred in FG1, FG2, FG3, FG5 and FG6; younger relatives also offered health-related advice.	FG5 included children requesting cultural and American foods. FG3 included shared decisions: “Once we agree, we cook it together” (FG3, P15). FG4 mainly documented food-provision roles.
Family roles schedules and meals	Preparation, storage, decisions and assistance organized food provision. FG1, FG3, FG4 and FG5 developed varied arrangements; FG2 and FG6 offered narrower coverage of this component.	“At the same time, not having to cook anjero every day is also a relief” (FG1, P1). FG4 described children providing food; FG5 described reliance on a particular cook.
Community and structural availability and affordability	FG1–FG6 distinguished obtaining familiar foods from affording them or finding preferred quality. Accounts included substitutions, multiple shops and assistance.	“Meat is available, but it is expensive, and I don’t know how fresh it is” (FG4, P24). FG2 described divergent retailer experiences; FG3 described community resources.
Community and structural access	FG1–FG6 described combinations of location, transport, acceptable products, information and assistance. Planning and help could make access manageable.	“My daughter does the grocery shopping for me, and I don’t have any barriers to getting food” (FG4, P25). FG5 contrasted manageable trips with reliance on others.
Community and structural migration stress activity and food exposure	Groups contributed different facets of dietary and perceived health change. Routine activity contrasts were explicit in FG1, FG2, FG5 and FG6; activity and sugar-exposure coverage was uneven.	FG6 included return from fast food to injera preparation. “For me, my health is actually better here” (FG3, P16) qualified uniform deterioration. Sugar practices did not establish increased intake.

## Data Availability

Our raw data analyzed in this study are available upon reasonable request due to privacy and ethical restrictions.. Requests will be reviewed in accordance with applicable data-sharing, ethical, privacy, or institutional requirements, and access will be provided where appropriate.
